# Sex Differences in Taxane Toxicities

**DOI:** 10.3390/cancers14143325

**Published:** 2022-07-08

**Authors:** Nicole N. Chmielewski, Charles L. Limoli

**Affiliations:** Department of Radiation Oncology, University of California, Irvine, CA 92697, USA; climoli@uci.edu

**Keywords:** taxanes, paclitaxel, sex differences, chemotherapy

## Abstract

**Simple Summary:**

Clinically observed sex differences in acute and long-term taxane chemotherapy-induced normal tissue toxicity are routinely documented but remain poorly understood despite the significant impact such toxicities have on treatment tolerance and quality of life outcomes in cancer survivors. This review draws from pre-clinical and clinical literature to highlight sex-specific mechanisms of action in taxane drug toxicity and proposes hypotheses for sex-specific clinical discrepancies in taxane-induced acute and long-term toxicities. To our knowledge, this is the first review exploring how sex as a biological variable impacts taxane-mediated mechanisms of action and clinical outcomes. In doing so, we have provided a novel framework to investigate and understand common sex differences observed in clinical and pre-clinical research.

**Abstract:**

The taxane family of microtubule poisons and chemotherapeutics have been studied for over 50 years and are among the most frequently used antineoplastic agents today. Still, limited research exists characterizing taxane-induced sex-specific mechanisms of action and toxicities in cancer and non-cancerous tissue. Such research is important to advance cancer treatment outcomes as well as to address clinically observed sex-differences in short- and long-term taxane-induced toxicities that have disproportionate effects on female and male cancer patients. To gain more insight into these underlying differences between the sexes, the following review draws from pre-clinical and clinical paclitaxel and taxane oncology literature, examines sex-discrepancies, and highlights uncharacterized sex-dependent mechanisms of action and clinical outcomes. To our knowledge, this is the first literature review to provide a current overview of the basic and clinical sex dimorphisms of taxane-induced effects. Most importantly, we hope to provide a starting point for improving and advancing sex-specific personalized chemotherapy and cancer treatment strategies as well as to present a novel approach to review sex as a biological variable in basic and clinical biology.

## 1. Introduction

The taxane family of chemotherapeutics are commonly used therapies for the treatment of hormone-refractory breast, prostate, and ovarian cancers, as well as lung and other cancers in women and men. Despite being the subject of scientific publications spanning over 50 years and among the most widely-used antineoplastic agents today, limited research exists characterizing taxane-induced sex-specific mechanisms of action and toxicities. Such research is important to provide insight into clinically-observed sex disparities in chemotherapeutic efficacies and adverse phenotypes [1,2,3,4,5,6,7,8], as well as providing further context into cancer incidence, susceptibility, and survivorship, where women typically have more favorable diagnoses and outcomes than men [7,9]. In addition, sex is still rarely considered when administering chemotherapeutic doses in the clinic [10]. In women, a chemotherapy dose is more frequently decreased during treatment compared to men due to greater acute normal tissue toxicity, such as nausea, vomiting, or neuropathic pain [11,12]. Increased sensitivity in women to chemotherapy’s adverse reactions is likely due to lower drug clearance rates compared to men [8,12]. These predictable pharmacokinetic sex discrepancies bring into question the validity of current body surface area chemotherapy dosing standards [13]. 

In order to gain more insight into sex differences from taxane therapy effects, the following review examines existing pre-clinical and clinical literature on taxanes in female and male models and highlights fundamental sex-dependent differences in mechanisms of action and clinical outcomes. To our knowledge, this is the first literature review summarizing sex differences in clinically relevant taxane toxicity phenotypes and provides a starting point, rather than a comprehensive analysis, for understanding, improving, and advancing personalized taxane chemotherapy and cancer treatment strategies for women and men. 

This review focuses on identifying sex differences in short- and long-term taxane-induced toxicities that affect quality-of-life (QOL), which is relevant to a growing population of cured and surviving patients [14]. Toxicities impacting QOL are of increasing concern to clinicians as aging cancer survivors are at the highest risk of developing long-term changes in cognitive function from exposure to non-specific therapies, such as cranial radiation and systemic chemotherapy [15,16,17,18]. Additionally, there are no known treatments to prevent or mitigate these effects, which often cause permanent changes in a variety of cognitive domains, spanning learning, memory, social behavior, and pain, and significantly impacting patient QOL. Female cancer survivors are the most susceptible population to long-term cancer treatment-induced cognitive dysfunction. This is in part due to higher survival rates for women compared to men with the same cancer diagnosis and quality of care [9], but also due to increased sensitivity to adverse chemotherapy toxicities [11,12]. Thus, there is a genuine need to further investigate the short- and long-term impacts of systemic anticancer therapies at the normal tissue level.

Drawing from paclitaxel and taxane oncology literature and our own research, we provide a current overview of the basic and clinical sex dimorphisms of taxane-induced effects and highlight promising new directions in the fields of taxane and sex differences research [19].

## 2. Paclitaxel and Taxane Background

Taxanes, starting with the discovery of paclitaxel in 1962, have an interesting and notable research and translational history that has been well reviewed [20,21,22,23,24]. In brief, paclitaxel, initially given the generic name of taxol upon isolation from the American Northwestern Pacific yew tree, *Taxus brevifolia Nutt.* (*Taxaceae*), was the only compound developed into clinical use from a 20-year collaborative effort of a nationwide plant-screening program for anticancer agents by the National Cancer Institute (NCI) and US Department of Agriculture [25]. The antineoplastic effects of paclitaxel were observed in animal cells in the 1970s [25,26,27,28], eventually leading to successful tumor regression in clinical trials by the 1980s [22,29]. Due to the limitations of public commercialization at the time, the NCI and US congress transferred the rights of paclitaxel from public to private property of Bristol-Myers Squibb in 1991, despite concerns over granting proprietary rights for a naturally-derived compound discovered through public funding [30]. The Federal Drug Administration (FDA) approved Bristol-Myers Squibb’s Taxol^®^ (generic term renamed to paclitaxel) as a chemotherapeutic treatment for ovarian cancer in 1992, breast cancer in 1994, and lung cancer in 1999 [31]. Paclitaxel has since become a frontline adjuvant treatment for many cancers and efficacy has led the way to the development of similar antimitotic compounds, known as the taxane family of chemotherapeutics. Of the family, docetaxel and cabazitaxel are also approved for clinical use, most notably for castration-resistant prostate cancers.

Taxanes are classified by their microtubule-stabilizing activity, inhibiting microtubule dynamics by preventing disassembly and cellular mitosis [25,28]. Taxanes are poorly soluble in water and require solvents to prevent crystallization during administration. Docetaxel is sufficiently dissolved in Tween 80 and ethanol, but paclitaxel requires Cremaphor EL, which is a powerful solvent known to induce adverse side-effects in patients through acute cytotoxicity. The novel nanoparticle albumin-bound formulation of paclitaxel (nab-paclitaxel) enables solubility in cells without Cremaphor EL. Semi-synthetic taxanes, docetaxel and cabazitaxel, were developed to address initial concerns over Pacific yew environmental protection as well as adverse reactions and resistance to paclitaxel neoadjuvant treatments [32]. Docetaxel, derived from the fast-growing European yew tree, *Taxus baccata*, was approved by the FDA in 1999 for breast cancer and in 2004 for prostate cancer. Docetaxel has a longer intracellular drug retention and greater affinity to β-tubulin than paclitaxel and differs in microtubule assembly effects and potency [33]. Cabazitaxel was developed to reduce the affinity to the ATP-binging cassette (ABC) drug efflux pump, MDR1/p-glycoprotein, which is a primary mechanism of resistance for paclitaxel and other chemotherapeutics [32]. Cabazitaxel is also more potent than paclitaxel and has shown effectiveness in docetaxel-resistant tumors. It was approved by the FDA in 2010 as a second-line treatment for docetaxel-resistant prostate cancer [34]. Although docetaxel and cabazitaxel have a higher potency than paclitaxel, paclitaxel is still the most commonly prescribed taxane. As a result, the majority of the literature reviewed studies paclitaxel, but the fundamental mechanisms of microtubule stabilization shared by all taxanes cause qualitatively similar effects and symptoms.

## 3. Translational and Clinical Research

Although little pre-clinical research exists comparing taxane sex effects, decades of clinical use documents sex-specific pharmacological differences after taxane chemotherapy administration. This is of particular concern for oncologists, as chemotherapy administration is based on body surface area instead of pharmacology and contributes to sex differences in therapy toxicities, which frequently lead to adjusted treatment doses [13]. This section will highlight the major sex-specific clinical observations during taxane treatments and corresponding pre-clinical literature that provide insight into the biological mechanisms behind sex differences. The section also covers basic pharmacokinetics as well as pharmacodynamically-focused adverse drug reactions and neurotoxicity, which account for taxanes’ dose-limiting toxicities and quality of life outcomes.

### 3.1. Pharmacokinetics

One of the most prominent sex differences in pharmacokinetics is the faster elimination of almost all chemotherapies (and many drugs) in men compared to women [3,8,35]. This is thought to reduce men’s risk of adverse drug reactions, but also brings to question whether each sex regularly receives their maximum tolerated dose, which is critical for optimizing curative intent [12,13,36]. A retrospective population analysis of solid tumor patients receiving paclitaxel infusions (*n* = 168) found that on average, male patients had a 20% higher maximal elimination capacity compared to female patients [37]. Paclitaxel is known to bind to plasma proteins extensively and non-specifically and, therefore, has negligible red blood cell transport [38], suggesting little impact of sex in hematocrit transport activity [39]. Sex differences in pharmacokinetics may impact drug disposition and important treatment factors, such as intracellular taxane concentrations in normal and metastatic tissue [40].

### 3.2. Adverse Drug Reactions

Clinical taxane use has been associated with more adverse drug reactions than other chemotherapies, particularly for breast cancer patients. The first group of breast cancer patients to receive paclitaxel (*n* = 55 studied) upon approval in 1999 demonstrated significantly higher rates of adverse acute toxicities compared to non-taxane-treated patients (*n* = 83), including arthralgia/myalgia (45% vs. 26%) and ataxia (20% vs. 5%) [41]. Paclitaxel-induced toxicities also seem to be more common in women, as one retrospective analysis of non-small lung cancer patients receiving paclitaxel reported more adverse drug reactions in women (77%) compared to men (66%, *p* = 0.0004). However, in these lung cancer patients, women demonstrated longer progression-free survival than men (Hazard Ratio (HR) 0.83, *p* = 0.02) [42]. Such data supports sex-specific trends in overall cancer toxicities as well as cancer outcomes [7]. Women are more likely to suffer from more serious adverse drug toxicities compared to men after most types of chemotherapy exposures [5,11,43], but benefit from better survival outcomes [44,45]. The current understanding of sex dimorphisms in disease suggest sex-specific immune modulation plays a critical role in cancer treatment-induced normal tissue toxicities and survival outcomes [46,47,48], thereby warranting further investigation into the context of taxane treatments.

The most acute taxane-associated adverse drug reactions are hypersensitivity reactions (HSRs), including flushing, hemodynamic alterations, dyspnea, musculoskeletal/neuropathic pain, and gastrointestinal issues [49]. All chemotherapeutics can induce HSRs varying in severity and degree, but taxane-induced HSRs typically manifest during the first or second infusion [50,51]. The immune response involving Immunoglobulin E (IgE) and/or IgG mast cell signaling mediates hypersensitivity reactions, but the impact of sex on mechanisms have yet to be characterized in pre-clinical or clinical literature. Fortunately, patients are successfully treated with a premedication of corticosteroids and antihistamines, but poor measures exist to predict patient risk, severity, and optimal treatment strategy for taxane-induced HSRs. This is in part due to the solvent and taxane moiety-dependent variability in HSR mechanisms, but sex has also been considered as a risk factor due to the pharmacokinetic differences of steroids and taxanes elimination rates and frequent observations of HSRs in breast and ovarian cancer patients [52]. In fact, a recent medical record analysis of 3181 Stanford Cancer Institute patients receiving paclitaxel or docetaxel associated the female sex with an increased risk of overall HSRs (HR 1.26) and gynecology oncology patients had an increased risk of overall (HR 1.34) and high-grade HSRs (HR 2.34) [53]. As mentioned before, such data support other trends of higher adverse toxicities in women. Since HSRs are easily treatable and transient (only associated with infusions), little pre-clinical research has explored sex-specific mechanisms. However, such research could provide critical insight into fundamental sex differences in the immune response that contributes to the development of other toxicities, such as taxane dose-limiting and quality of life-impairing neurotoxicity.

### 3.3. Neurotoxicity

Most chemotherapeutic agents, including taxanes, have limited blood brain barrier (BBB) penetration and are readily purged from the brain through P-glycoprotein (P-gp) pumps [54,55]. However, certain taxane preparations, like cabazitaxel, have greater BBB permeability [56] or contain solvents associated with additional BBB permeability, such as paclitaxel in Cremophor EL, which inhibits hippocampal cell proliferation in rodents [57]. Dorsal root ganglia and peripheral nerves are subject to chemotherapeutic agent toxicity through blood–nerve barrier permeability, which, due to lymphatic and P-gp absence in nerves, likely causes the commonly observed chemotherapy-induced peripheral neuropathic toxicities [58]. Although such distinguishing features of the central nervous system are thought to exist independent of sex, the majority of such basic research has utilized male rodents and therefore sex differences in the aforementioned neuronal characteristics cannot be entirely ruled out.

Clinical manifestations of taxane-induced neurotoxicity predominantly include peripheral neuropathy and cognitive dysfunction [59]. Limited comparisons of sex differences in the neurotoxicity of taxane recipients exist, but as most clinical data document women (predominantly breast cancer survivors), broader comparisons are confounded and suggest the preponderance of taxane-induced neurotoxic phenotypes that burden women.

#### 3.3.1. Peripheral Neuropathy

Taxane-induced peripheral neuropathy (PN)-associated pain is the most concerning and critical clinical observation affecting patients’ immediate and long-term quality of life [59,60,61,62]. Since the elimination of chemotherapy-induced neutropenia through the implementation of preventative granulocyte colony-stimulating factor treatment [63], PN-associated pain is the most common reason for taxane treatment dose reductions [64]. Although most cancer patients receiving taxanes develop PN [59], no comprehensive analysis of clinically observed sex differences exists. In an analysis of 219 American breast cancer survivors treated with adjuvant paclitaxel, 97% developed PN and 60% developed chronic PN one year following the cessation of treatment cessation [65]. On the other hand, in 82 prostate cancer patients who received docetaxel, only 32% developed PN [66]. Although docetaxel is thought to induce less PN than paclitaxel [59], these data further highlight the distinct clinical sex differences in taxane treatment strategies and PN outcomes that produce a greater burden on female cancer survivors [67,68]. Moreover, only non-pharmacological PN pain management strategies exist for taxane recipients [59,69,70]. One reason for unsuccessful pharmacological approaches may be due to the lack of mechanistic insight regarding fundamental sex differences in taxane-induced PN. However, in recent years, growing pre-clinical evidence suggests that taxane-induced PN manifests through distinct neuroimmune mechanisms in female and male rodents and may provide valuable insight into understanding and treating sex differences in taxane-induced PN as well as other toxicities.

Based on pre-clinical studies primarily using male models [71], glial cells and pro-inflammatory immune responses from innate immune cells are implicated in peripheral neuropathy [72], including taxane-induced pain [73]. Candidate mechanisms, such as toll-like receptor (TLR) 4 activation [74], which is extensively studied in male rodents, have only recently been examined in females. As a result, sex-specific findings in the field of pain have been categorical, illustrating that microglia, and their associated signaling molecules, drive neuropathic pain in only the male sex [75,76,77].

Supporting this, one recent study found that TLR9 inhibition only attenuate paclitaxel-induced mechanical allodynia (touch induced pain) in males and not female mice. However, interestingly, TLR9 antagonism reduced paclitaxel-induced pain in female nude mice lacking T and B cells [78], suggesting that females preferentially utilize humoral/adaptive immunity, but also retain the ability to recruit innate immunity-dependent microglia when adaptive immunity is unavailable [77].

Sex hormones and sex organs drive sex-specific immunity [46]. In one study examining pain thresholds and pro-inflammatory cytokine receptor levels in the dorsal root ganglion of female rats exposed to paclitaxel, ovariectomies significantly increased pain thresholds and decreased receptor expression levels after exposure compared to females with 17β-estradiol and progesterone replacement [79], suggesting that paclitaxel-induced neuropathic pain in females is sex hormone-dependent.

Sex differences in stress phenotypes [80] is a growing field of research as well as a clinical risk factor and comorbidity of taxane-induced PN [68]. In a study examining the neuroendocrine stress axis in rats, paclitaxel-induced hyperalgesia was significantly more attenuated in female rats compared to males following β2-adrenergic receptor reduction through targeted intrathecal antisense oligodeoxynucleotide administration (ODN). By contrast, ODN decreasing glucocorticoid receptors attenuated paclitaxel-induced pain in males, but not females. Additionally, the study found that neonatal handling prevented paclitaxel-induced PN in male but not female rats, providing evidence of distinct sex-specific neuroendocrine mechanisms related to paclitaxel-induced pain [81].

Based on these recent findings and the current understanding of sex differences in immune functions, female-specific pain is likely due to humoral immune responses, such as T and B cell activation, and may require novel treatment approaches and a more sophisticated understanding of mechanisms in both sexes [82,83,84]. These data also bring into question the other possible impacts of sexually dimorphic immune responses in taxane-induced toxicities. Divergent preferential immune function between the sexes, defined as females utilizing more adaptive immunity while males rely more heavily on innate immunity, is becoming a dominant theme in pharmacology and etiology [7,46,85] and, therefore, perhaps it should be the starting point of studying sex as a biological variable in drug and disease research.

#### 3.3.2. Cognitive Dysfunction

Chemotherapy-induced cognitive dysfunction, also known as ‘chemobrain,’ is of growing clinical concern due to detrimental impacts on long-term quality of life in increasing populations of aging cancer survivors [16,86,87,88,89,90,91,92]. As outlined in the above Peripheral Neuropathy section, the existing literature of pre-clinical rodent studies comparing both sexes suggest greater taxane-induced peripheral neuropathic pain in females than males. However, paradoxically, recent (albeit limited) literature comparing paclitaxel-induced cognitive dysfunction in both sexes demonstrates female-specific resistance. Sixteen mg/kg of paclitaxel (dissolved in Cremophor EL and ethanol) in intraperitoneal exposures of C57BL6 mice induced cognitive dysfunction in a prefrontal cortex-associated Novel Object Recognition task and anxiety-like behavior in Elevated Plus Maze in males but not females [93]. We also observed female-specific cognitive and cytotoxicity protection after 150 mg/kg and 300 mg/kg paclitaxel (dissolved in Saline and ethanol) exposures in C57BL6 mice, characterized by significantly less weight loss, higher survival, sustained prefrontal cortex-associated Fear Extinction performance, and less anxiety-like behavior during Light/Dark Box testing in female rodents [94]. In the same study, we hypothesized that (a) reproductive senescence in aged Wild-Type females and (b) RhoB GTPase deficiency would attenuate the female-specific resistance through short-circuiting estrogen-mediated female-specific (a) neuroprotection [95,96] and (b) RhoB-dependent protection [97]. Interestingly, aged (22-month-old) female Wild-Type and female and male RhoB-deficient animals were all resistant from paclitaxel-induced cognitive dysfunction, but as hypothesized, survival and weight decreases occurred in aged Wild-Type and RhoB-deficient females. Such data demonstrating female-specific neuroprotection seem contrary to the cognitive impairment frequently reported with adjuvant taxane treatments in women.

The first group of breast cancer patients to receive the FDA-approved paclitaxel (*n* = 55 studied) not only demonstrated significantly higher rates of adverse acute toxicities (mentioned in the Adverse Drug Reactions section), but also significantly more mental distress and reduced mental quality of life compared to women who received other chemotherapies (*p* < 0.023). In addition, this study observed longer emotional recovery for paclitaxel-treated patients, which required an average of 2 years compared to 6–12 months for patients not receiving taxanes [41]. In a recently published study comparing sexes, a small population of nasopharyngeal carcinoma survivors receiving adjuvant docetaxel, cisplatin, and fluorouracil chemotherapy, found that the female sex was associated with cognitive dysfunction (*p* = 0.039), manifesting in 50% of women (8 of *n* = 16) and only 20% of men (10 of *n* = 50) [98]. A 2017 longitudinal study of breast cancer survivors comparing adjuvant chemotherapies with and without taxanes (*n* = 51) demonstrated short- and long-term cognitive impairment in attention and executive function after all treatments, with a more pronounced impact on short-term verbal learning and speed measures in the taxane group [99]. Another analysis of breast cancer survivors shortly after and 1 year after adjuvant chemotherapy (majority with paclitaxel, up to 8 patients without) demonstrated acute (65%, 24 of *n* = 37) and long-term (61%, 17 of *n* = 28) cognitive decline [100]. A recent female-only rodent study supports these clinical data of adjuvant therapy-induced cognitive impairment in women. Brown et al. characterized the diminished hippocampal-dependent cognitive behavior and compromised dendritic architecture and signaling proteins in female hippocampal tissue after adjuvant docetaxel, doxorubicin, and cyclophosphamide therapy [101].

In perhaps the only clinical analysis of cognitive functioning after taxanes in males, 65+ year old castrate-resistant prostate cancer patients treated with adjuvant therapies, including docetaxel, did not experience significant cognitive impairment measured by the Montreal Cognitive Assessment [102]. In contrast, an analysis comparing 65+ year old early-stage breast cancer survivors of all treatments (radiotherapy with or without doxorubicin ± docetaxel) demonstrated significant impacts on cognitive decline in 49% of patients, with the oldest patients (70–81 years) being most sensitive to docetaxel-associated decline (*p* = 0.05) [103].

Although little research investigates sex-specific burdens of chemotherapy-induced cognitive dysfunction in cancer survivors, it is interesting to point out that almost all clinical studies assessing taxane-induced cognitive outcomes are in breast cancer survivors, while most pre-clinical rodent studies examine outcomes in males [57,104,105,106,107]. The larger amount of female-specific clinical data is in part due to the total of 8.7 million women cancer survivors in the U.S. (over 600,000 more survivors than men), of which 3.8 million are breast cancer survivors [44]. In addition, the larger proportions of women suffering from adverse chemotherapy effects compared to men undoubtably impacts stress and depression and, therefore, cognitive function and quality of life during and after recovery. The diversity in clinical patient data (e.g., cancer type, adjuvant treatments, age, etc.) is not always comparable to highly controlled pre-clinical studies and likely accounts for the discrepancy in taxane-induced cognitive dysfunction phenotypes presenting in women, but less frequently in female rodents.

## 4. Basic Research

‘Mechanism of action’ in cancer therapy refers to an agent’s primary antineoplastic mechanism, which is often antimitotic in nature. Twentieth century chemotherapy pre-clinical research almost exclusively studied cancer cells, and scientists were predominantly concerned with the ‘mechanisms of action’ directly related to inhibiting cancer growth. In the 21st century, as survival rates and treatment efficacies increased, quality-of-life outcomes became more important and a growing issue for cancer survivors, increasing biomedical research interests in normal tissue effects. Due to the non-specific nature of chemotherapeutics, it is important to consider how mechanisms of action impact normal tissue toxicity and sex.

It is interesting to note that virtually all basic and pre-clinical in vivo cancer drug research done in the 1900s, such as with paclitaxel, utilized female HeLa cervical adenocarcinoma cell lines, the first and most widely used immortalized human cell line derived from 31-year-old Henrietta Lacks’ aggressive cervical cancer in 1951 [25,108]. Although no other cell line has contributed more to foundational biological understanding and biomedical advances, its sex was rarely considered or examined as an experimental variable. Despite little consideration of sex in basic research, clinical data provides justification for such research. As discussed earlier in Translational and Clinical Research, women have better treatment outcomes across most cancer types, and sex differences are readily observed in adverse drug effects, drug disposition, and pharmacokinetics, which undoubtably impacts chemotherapy antineoplastic efficacy and normal tissue toxicity. The following sections draw from fundamental research (not considering sex) of taxane antineoplastic activity and sex-specific cancer data to highlight potential sex-specific mechanisms of toxicity relevant to most, if not all, tissue.

### 4.1. Mitotic Arrest

**Figure 1:**
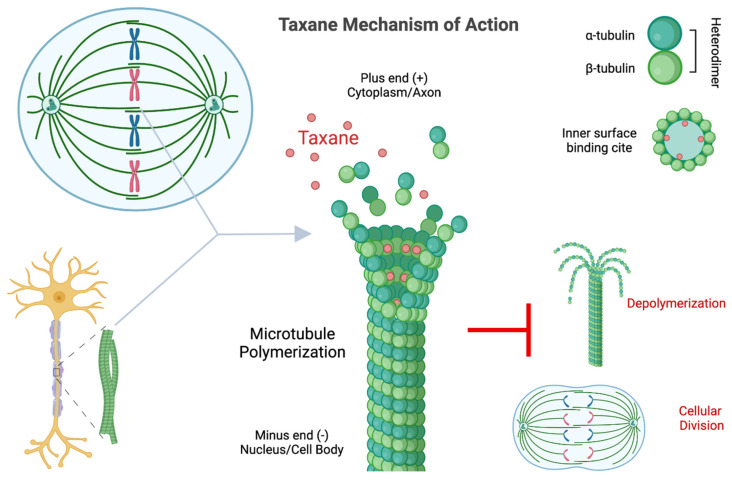
Known taxane mechanism of action. Taxanes bind to the inner surface of microtubule filaments, on the β-tubulin globular protein of the heterodimer, and the protofilament tubule arrangement. Taxanes prevent microtubule depolymerization, preventing normal microtubule dynamics for cellular activities and impacting cytoskeletal structure and microtubule function, such as mitosis and microtubule-dependent cellular transport. Created with BioRender.

After initial observations of paclitaxel-induced Kb HeLa-derived cell growth inhibition, published in 1971 [25], tumor drug researcher, Susan Horwitz, was the first to characterize the mechanism of paclitaxel-induced mitotic arrest with her graduate student, Peter Schiff [24]. Their landmark 1979 publication identified paclitaxel as a microtubule assembly promoting poison, unlike previously identified microtubule poisons that prevent microtubule polymerization. In the words of Horwitz, microtubules behave like “paralyzed cytoskeleton[s]” [109] in the presence of β-tubulin-bound paclitaxel. Paclitaxel reduces the critical concentration of tubulin subunits necessary for microtubule polymer formation, increasing the percentage of α- and β-tubulin heterodimers assembled, and inhibits mitosis through the failure of metaphase depolymerization. They observed that paclitaxel-induced microtubule growth was even resistant to cold and calcium depolymerization treatments [28]. The observation of paclitaxel-induced polymerization and the “parlay[sis]” of microtubules causing mitotic arrest was confirmed in a variety of cell and animal models, in both cancerous and non-cancerous tissue [27,110,111,112]. For decades, the dominant perception of paclitaxel’s tumor treatment efficacy was through the inhibition of metaphase bipolar spindle depolymerization and mitotic checkpoint-dependent mitotic arrest [113,114]. However, investigators have also observed taxane-induced apoptosis that is unassociated with mitotic arrest with formidable cancer regression in low-proliferating tumors, suggesting alternative cytotoxic pathways that are critical in taxane antineoplastic activity [111,112,115,116,117]. In fact, drugs designed to exclusively inhibit mitosis have shown limited antineoplastic success and fail to replace microtubule poison chemotherapeutics [111,116,118,119]. Advances in characterizing taxane-induced microtubule dysfunction beyond mitotic arrest suggest alternative cytotoxic mechanisms of action may be the preponderance of taxane anticancer activity (Figure 1).

### 4.2. Microtubule Dynamic Dysfunction

Although the primary antineoplastic mechanism of taxanes was initially thought to involve mitotic arrest through microtubule dysfunction, current scientific consensus also attributes taxane tumoral regression efficacy to additional, perhaps more potent, microtubule-dependent cytotoxic mechanisms (refer to prior section on Mitotic Arrest). Due to the importance of microtubule function in a range of cellular activity, most notably mitotic spindle assembly, cytoskeletal structure, and cytoplasmic cellular cargo migration [120,121], it is hypothesized that taxanes also elicit antineoplastic activity through mitotic checkpoint-independent cell death [115,117,122] and cellular transport disruption [123,124,125]. In fact, early paclitaxel research demonstrated aberrant microtubule dynamics and toxicity at doses insufficient to induce mitotic arrest [126,127,128,129]. In a notable recent study, adjuvant paclitaxel-treated human breast tumor biopsies revealed that intratumoral drug concentrations were insufficient to induce mitotic arrest and tumor regression efficacy, which was attributed to increased multipolar spindle formations, implicating p53-independent, chromosome missegregation-induced cellular death [122]. Such data suggest that taxanes can produce a range of microtubule-dependent aberrant phenotypes, dependent on the intracellular drug concentration and phase of cell cycle.

Understanding the impact of taxanes on microtubule-dependent nuclear trafficking and signaling is perhaps one of the most challenging phenotypes to characterize due to the extent and complexity of microtubule-mediated mechanisms. However, the influence of taxanes on androgen receptor signaling has been extensively observed and studied in the field of prostate cancer and provides additional understanding about the impact of taxane-induced microtubule dysfunction and sex-specific mechanisms of toxicity.

#### 4.2.1. Androgen Receptor Signaling Dysfunction

**Figure 2:**
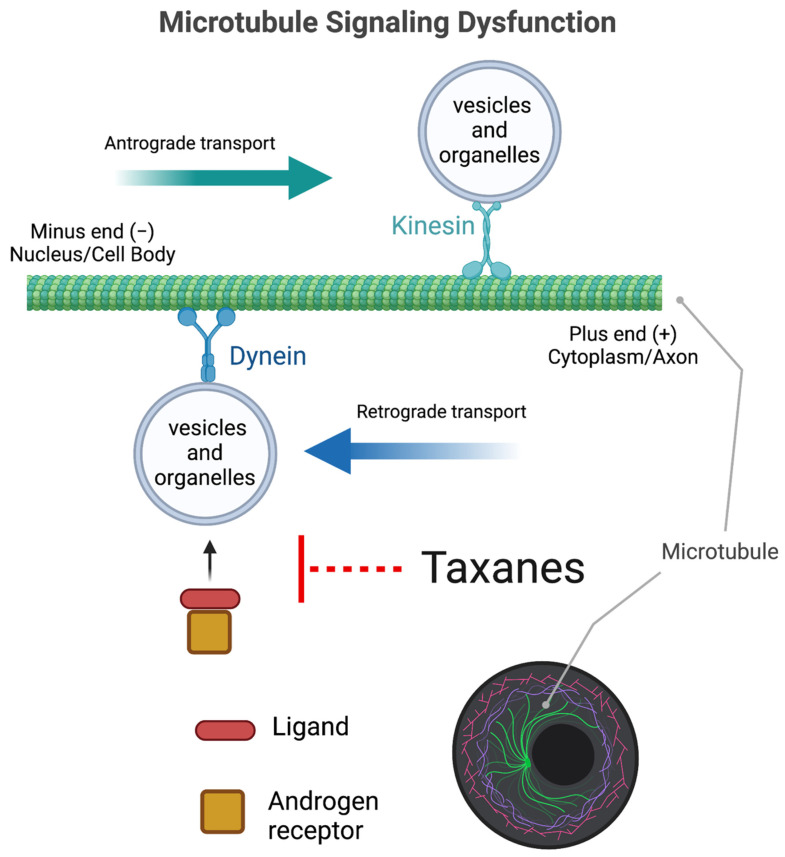
Taxane-induced androgen receptor (AR) microtubule-dependent signaling dysfunction. In the field of prostate cancer research, taxanes are observed to interfere with ligand-mediated AR signaling by inhibiting AR binding to dynein motor proteins, preventing nuclear trafficking of AR. Created with BioRender.

In the field of prostate cancer research, taxanes are known to inhibit androgen receptor (AR) signaling through microtubule dysfunction [125,130] (Figure 2). AR signaling mechanisms are the cause and treatment target for prostate cancers [131,132,133]. Androgen deprivation therapy (ADT), which is used to block AR or eliminate AR ligands, initially works to inhibit the growth of prostate cancers as a frontline treatment, but over time castrate-resistant prostate cancer (CRPC) occurs through loss of ADT sensitivity [132]. In 2004, taxanes became the first class of chemotherapeutic drugs that demonstrated improved survival in CRPC and are now used in conjunction with frontline ADT treatments prior to the development of castration resistance [134,135,136,137]. Although research on the effects of taxanes on AR signaling is almost exclusive to the field of prostate oncology, these male-specific observations provide context into fundamental sex differences of systemic taxane exposures for male and female malignant and normal tissue. 

Researchers have observed the taxane-dependent inhibition of AR expression and activity in prostate cancer [130,138,139]. Specifically, Darshan et al. demonstrated that paclitaxel inhibits microtubule ligand-induced AR nuclear accumulation and downstream transcriptional activity [125]. The same study indicated a significant correlation between AR cytoplasmic sequestration (instead of nuclear accumulation) and therapeutic efficacy in the circulating tumor cells of CRPC patients receiving taxanes. Interestingly, although not entirely understood, taxane-induced interference with AR signaling produces antineoplastic efficacy in both naïve and castrate-resistant malignancies [132]. One pre-clinical research study observed that chronic AR activation, through testosterone-BSA exposure, enhanced paclitaxel microtubule disrupting dynamics, inhibited cell proliferation, and induced apoptosis in androgen sensitive and insensitive human prostate cancer cell lines [140]. In the same study, xenografted mouse tumors (both androgen sensitive and insensitive) decreased in mass with both testosterone-BSA and paclitaxel separately, an effect that was enhanced in combination. These data support clinical observations of taxane treatment efficacies in prostate cancers that are sensitive and resistant to ADT [134].

Androgen receptor signaling is critical for prostate cancer as well as normal male tissue development, thus providing a potential sex-specific discrepancy in taxane tissue toxicity. AR knockout (KO) mouse studies have not only demonstrated the critical function of AR in male (and female) gonad and gamete development, but also in male body fat, bone, blood, and immune phenotypes [141,142,143,144]. Conditional and constitutive ARKO male mice have severe deficits in both innate and adaptive immunity, resulting in phenotypes such as thymus enlargement, immature B-cell populations, and risk of neutropenia and bacterial infections [141]. Although little research has investigated such ARKO phenotypes in female mice, since ARs dominate signaling function for male sex hormones (while estrogen receptors dominate female sex hormones), taxanes may induce disproportionate toxicity in male tissue. Supporting this idea, a developmentally critical Y-linked gene (found in male mice and men) modulates docetaxel sensitivity through AR signaling [145]. KDM5D (lysine-specific demethylase 5D) physically interacts with nuclear AR and demethylates (deactivates) H3K4me3 transcriptional marks, which normally regulate enhanced AR transcriptional activity (possibly via negative feedback). Accordingly, the attenuation of KDM5D expression led to increases in H3H4me3 marks in promoter regions of AR-regulated genes as well as protection against docetaxel in AR-positive prostate cancer cell lines. In addition, an Oncomine cancer database analysis revealed significantly lower KDM5D expression in CRPC patient samples with a poorer cancer prognosis and treatment outcome. Although KDM5D expression is associated with taxane sensitivity, the fundamental mechanism is still unclear. ERG (E-26 transformation-specific-related gene) is another gene implicated in AR-signaling in prostate cancer [146,147] through microtubule depolymerization. ERG binds to αβ-tubulin, and when overexpressed, reduces binding site availability for taxane-induced microtubule polymerization, leading to taxane resistance in prostate cancer cells and tumors [148]. Such data defines taxane as AR signaling poisons, in part, due to epigenetic regulation. However, it is important to note that AR signaling can also take place through nuclear transport in a microtubule-independent fashion. The capability of tumors to preferentially shuttle AR through nuclear transport is thought to contribute to taxane resistance in prostate cancer. The ability to circumvent microtubule transport machinery has been demonstrated by AR-V7, which is a commonly found AR splice variant that lacks the hinging domain necessary to attach to the tubulin-dynein transporter molecule for minus-end (nuclear) microtubule transport [149]. The identification of AR-V7 splice variants in tumors is associated with advanced CRPC, taxane resistance, and reduced patient survival [150,151,152,153,154,155].

#### 4.2.2. Estrogen-Mediated Microtubule Dynamics

As the field of prostate cancer continues to elucidate the complex relationships between taxane-induced microtubule dysfunction and AR signaling, the role of estrogen and estrogen receptors (ER) on taxane-induced microtubule dynamics is less clear. However, a couple of studies suggest that estrogens and androgens may have opposite roles in microtubule polymerization. One study examining the roles of sex hormone exposures on tubulin polymerization induction observed that prior incubation of tubulin proteins and cells, derived from fetal rat hippocampi, with 17β-estradiol inhibited microtubule assembly, while incubation with testosterone inhibited microtubule disassembly [156]. Other studies have also observed an AR+ dependent relationship between testosterone/androgen and microtubule polymerization [140,157], suggesting the presence of androgens may promote microtubule polymerization/assembly indirectly. On the other hand, recent evidence suggests estrogens may have a direct role in microtubule polymerization and function. Lo et al. conducted a computational protein screen analysis that unexpectedly identified ER as a cognate receptor to the β-tubulin taxane binding site, suggesting tubulin–ER cross-reactivity. The researchers then confirmed that the taxane binding site had affinity with estrogen and selective estrogen receptor modulators (SERMs) in human epithelial cells and modified microtubule dynamics in a similar fashion as paclitaxel [158]. Interestingly, a separate in vitro study observed no synergistic or additive antimetastatic effects with the co-administration of the SERM, tamoxifen, with paclitaxel in ER+ breast cancer cells [159]. Such data suggests that ER ligands may directly interact with tubulin and modulate microtubule dynamics, and in the presence of taxanes, attenuate taxane-induced microtubule polymerization.

In summary, the foregoing sections outline possible sex-hormone-mediated sex differences in taxane-dependent microtubule dysfunction. Taxanes induce AR signaling dysfunction in a microtubule dependent manner that provide a sex-specific toxicity in male tissue due to androgen dominance. In females, the cross-reactivity of ER ligands at the taxane-tubulin binding site may inhibit taxane-mediated microtubule polymerization and attenuate downstream microtubule-dependent toxicity. This suggests sex-hormone-mediated male-specific susceptibility and female-specific protection against taxane-induced microtubule dysfunction. Our recent study, observing significant male-specific normal tissue cytotoxicity after paclitaxel exposure in mice [94], provides further evidence of sex-specific taxane-induced toxicity and highlights the importance of investigating normal tissue effects of systemic cancer treatment.

## 5. Conclusions

This review provides the first multidisciplinary analysis highlighting how basic sex-specific taxane-induced toxicity mechanisms may impact clinical taxane treatment outcomes. Basic research has established microtubule dynamic dysfunction as the primary antineoplastic mechanism of action through the inhibition of mitosis and other microtubule-dependent cellular functions. Taxane-induced microtubule dependent androgen receptor signaling dysfunction (extensively studied in the field of prostate cancer) and the potential cross-reactivity of estrogen receptor ligands inhibiting taxane-tubulin binding provides the most robust evidence for developing a hypothesis for male-specific toxicity and female-specific protection after paclitaxel exposure. However, clinical observations paradoxically show higher risk in female patients during and after taxane treatment for developing acute and long-term adverse effects. Considering both basic mechanisms and clinical observations, sex hormones (androgens and estrogens), adjuvant exposure strategies, and their cumulative impacts on sex-specific immune modulation likely account for this discrepancy.

Despite the defined mechanism of action of taxanes on the inhibition of microtubule depolymerization and dynamics, other downstream effects of microtubule dysfunction on cellular activities, whether mitosis, intracellular transport, or cytoskeletal dynamics, may well engage immune response pathways. Accordingly, mounting evidence highlights sexually dimorphic strategies of immune responses, which are mediated by sex hormones and sex organs [46,47,83,85,160]. As mentioned in the Section 3.3.1, the field of pain research, including taxane-induced peripheral neuropathy, has characterized significant sex differences in the last decade [75,76,77]. Females manifest neuropathic pain through humoral response and adaptive immunity, while pain in males is induced through innate immune response [77]. Other fields have characterized similar trends in sex-specific systemic immunity, such as in infectious disease research (e.g., SARS-CoV-2) [161,162,163,164,165] and in chronic autoimmune conditions [46,47,48,85,160]. Therefore, although males are more susceptible to taxane-induced microtubule-dependent dysfunction through reliance on AR signaling, they may be protected from acute and chronic humoral responses initiated by autoimmune dysfunction, which are characteristic of hypersensitivity reactions, nausea, vomiting, and peripheral neuropathy more commonly found in female patients treated with taxanes. Although women are susceptible to greater normal tissue discomfort and toxicity from taxanes, they are also likely to benefit from greater antineoplastic efficacy, potentially accounting for better survival outcomes in female patients.

This review highlights the importance of addressing sex as a biological variable in research and provides a framework to investigate sex differences in other fields of drug and disease research. In the case of taxane research, although the field of prostate cancer clearly identifies androgen receptor signaling as a biological target for taxanes, sex comparisons do not exist, and sex-specific toxicities still remain under-investigated. In the case of peripheral neuropathy research, the recent addition of female models has shifted the fundamental understanding of pain mechanisms and strategies to incorporate sex as an important treatment variable. In sum, pre-clinical studies focused on sex differences remain understudied and are essential to not only decipher clinical observations, but to also compare fundamental biological mechanisms of action for the purpose of advancing personalized treatment strategies and improving the quality of life of male and female cancer survivors alike.
